# Ebola disease outbreak caused by Bundibugyo virus in Africa: A defining moment for Africa’s health security and sovereignty

**DOI:** 10.4102/jphia.v17i1.2069

**Published:** 2026-06-02

**Authors:** Yap Boum, Nebiyu Dereje, Kyeng Mercy, Mosoka Fallah, Merawi Aragaw, Landry Tsague, Raji Tajudeen, Shanelle Hall, Ngashi Ngongo, Jean Kaseya

**Affiliations:** 1 Africa Centres for Disease Control and Prevention, Addis Ababa, Ethiopia

The ongoing Bundibugyo Ebola virus disease outbreak affecting the Democratic Republic of the Congo (DRC) and Uganda represents one of the most complex epidemic emergencies Africa has faced in recent years. Unlike previous Ebola outbreaks largely dominated by the Zaire strain, the current outbreak involves Bundibugyo ebolavirus, a rare species for which validated vaccines, therapeutics and diagnostics remain extremely limited.^1^ Beyond its epidemiological significance, however, this outbreak is emerging as a defining opportunity to accelerate the operationalisation of Africa’s Health Security and Sovereignty (AHSS) agenda.^2^

The outbreak is unfolding in exceptionally difficult operational conditions. As of May 2026, active transmission is occurring in Ituri and North Kivu provinces, insecure and conflict-affected areas of eastern DRC characterised by armed groups, weak health systems, intense mining-related mobility, population displacement and limited access to healthcare services. Uganda has already confirmed imported cases linked to travel from the DRC, while South Sudan and neighbouring countries remain at elevated risk due to intense cross-border population movement. Community deaths outside formal health structures, delayed detection and gaps in infection prevention and control (IPC) have increased the risk of sustained transmission and regional amplification.^3^

Yet, despite these challenges, the outbreak is testing the growing maturity of Africa’s continental health security architecture. Following extensive review by the Africa Centres for Disease Control and Prevention (Africa CDC) Emergency Consultative Group (ECG), composed of independent experts, Africa CDC declared the outbreak a Public Health Emergency of Continental Security (PHECS). The decision reflected a structured scientific process grounded in epidemiological evidence, operational realities, regional risks and preparedness capacities. The ECG unanimously concluded that the outbreak met the threshold criteria for a PHECS because of its severity, cross-border transmission risks, operational complexity, limited medical countermeasures, and implications for regional and global health security.^3^

Most importantly, this declaration demonstrates the increasing role of African leadership in shaping the governance of health emergencies on the continent. Extensive consultations were held with African Union leadership, affected Member States and regional stakeholders before the declaration. This reflects the emergence of a continental policy where Africa leads and multilateral organisations support, underscoring the way forward to reshaping the global health architecture.^4^ The strong political commitment shown by African leaders to empower the Africa CDC to declare a PHECS and mobilise continental solidarity represents a major shift towards African ownership of health security governance.

The current outbreak is a key opportunity to operationalise the five strategic pillars of the AHSS agenda: reform global health architecture, institutionalise pandemic prevention, preparedness and response (PPPR), digital transformation, local manufacturing and health financing.

Firstly, the outbreak demonstrates the importance of domestic financing and African-owned emergency response mechanisms. Africa CDC, the African Development Bank and African countries have begun allocating funds to support response efforts, representing a significant shift from historical reliance on delayed external emergency financing towards African-led, rapid resource mobilisation.

Secondly, the outbreak is accelerating operationalisation of the PPPR pillar through deployment of the Emergency Operational Response System (EORS). Within 72 h following the outbreak confirmation in DRC and Uganda, Africa CDC deployed technical experts and African Volunteer Health Corps (AVoHC) responders to support surveillance, laboratory systems, coordination, Risk Communication and Community Engagement (RCCE), IPC and cross-border preparedness activities. This rapid deployment mechanism directly contributes to the 7-1-7 target for outbreak detection, notification and response.

Thirdly, digital transformation is becoming central to the response strategy.^5^ Building on lessons learned during the mpox outbreak, Africa CDC supports the deployment of community health workers equipped with Android-based tools connected to DHIS2 Tracker systems to strengthen real-time community-based surveillance. Community surveillance remains one of the five most critical pillars of successful outbreak control, particularly in fragile and insecure settings where formal health systems are limited. The integration of digital surveillance, real-time data analytics and decentralised reporting systems is expected to significantly improve early detection, contact tracing and operational coordination to control the outbreak despite the delayed detection.

Fourthly, the outbreak highlights the strategic importance of African local manufacturing capacities for medical countermeasures.^6^ One of the major operational gaps remains access to diagnostics for Bundibugyo ebolavirus and other emerging pathogens. Africa CDC is supporting discussions around scaling the production of the Radione multiplex diagnostic platform in Africa, with an estimated investment need of US$2 million – US$3 million. Importantly, this platform, initially deployed in DRC as part of the mpox response legacy, is capable of decentralised detection of mpox, measles, varicella, cholera and all Ebola virus species, including Bundibugyo strains. This represents a transformative opportunity to move beyond emergency procurement towards sustainable African manufacturing ecosystems that can support epidemic preparedness and response.

Fifthly, the outbreak is reinforcing ongoing efforts to transform the global health architecture.^7^ Discussions around equitable financing, local manufacturing, emergency coordination and African leadership were central during the recent World Health Assembly, shortly before the Africa CDC delegation led by its Director General returned to Africa to coordinate the ongoing Ebola response. The rapid activation of continental coordination systems through the Incident Management Support Team (IMST), jointly coordinated with WHO under the ‘4 Ones’ principle – One Team, One Plan, One Budget, and One Monitoring & Evaluation Framework – demonstrates that Africa is increasingly developing operational models capable of coordinating complex emergencies across multiple countries.^8,9,10^

The outbreak simultaneously exposes persistent inequities in global epidemic preparedness systems. Substantial investments have been made globally for vaccines and therapeutics targeting Zaire ebolavirus, yet far less attention has been devoted to Bundibugyo and other Ebola species. Africa is once again confronting a severe outbreak with limited validated vaccines, therapeutics and diagnostics. This inequity reinforces the urgency of strengthening African-led research, genomic surveillance, operational science, local manufacturing and clinical trial ecosystems capable of addressing pathogens most relevant to African epidemiology.^1,11,12^

Importantly, the declaration of a PHECS should not trigger unnecessary border closures or punitive travel restrictions. Previous outbreaks repeatedly demonstrated that blanket border closures undermine surveillance, disrupt humanitarian and economic activities, fuel informal movement, and weaken trust between communities and authorities. Instead, Africa CDC and partners are prioritising strengthened screening at Points of Entry, cross-border surveillance, laboratory readiness and preparedness activities while maintaining coordinated regional mobility.

Ultimately, the Bundibugyo Ebola outbreak may be remembered not only for its epidemiological complexity, but also for accelerating the transformation of the Africa Health Security and Sovereignty’s agenda from vision to implementation. The operationalisation of the PHECS mechanism, the rapid deployment of EORS, the strengthening of digital surveillance systems, the advancement of local manufacturing discussions, the mobilisation of African financing, and the consolidation of the IMST coordination model collectively demonstrate the growing maturity of African leadership in public health emergency governance.

Africa is increasingly showing that continental institutions can lead science-based emergency responses while multilateral organisations provide support within an African-led framework. In doing so, the continent is not only strengthening its own health sovereignty but also contributing directly to the transformation of global health architecture.
